# Diagnostic and therapeutic delays in lung cancer during the COVID-19 pandemic: a single center experience at a German Cancer center

**DOI:** 10.1186/s12890-024-03082-x

**Published:** 2024-07-04

**Authors:** Thomas S. Tarawneh, Elisabeth K. M. Mack, Charis Faoro, Andreas Neubauer, Martin Middeke, Andreas Kirschbaum, Angélique Holland

**Affiliations:** 1 Department of Hematology, Oncology and Immunology, Philipps-University Marburg, 35043 Baldingerstraße, Marburg, Germany; 2 Comprehensive Cancer Center Marburg, Philipps-University Marburg, 35043 Baldingerstraße, Marburg, Germany; 3 Department of Visceral, Thoracic and Vascular Surgery, Philipps-University Marburg, 35043 Baldingerstraße, Marburg, Germany; 4 Department of Pulmonary and Critical Care Medicine, Philipps-University Marburg, 35043 Baldingerstraße, Marburg, Germany

**Keywords:** COVID-19, Sars-CoV-2, Lung cancer, Cancer staging, Screening, Diagnosis

## Abstract

**Background:**

The COVID-19 pandemic has had negative drawbacks on the healthcare system worldwide and on individuals other than those directly affected by the virus. Delays in cancer therapy and diagnosis have been reported in the literature. We hypothesized similar effects on patients with lung cancer at our center.

**Methods:**

We retrospectively analyzed data of patients referred to our center with newly diagnosed lung cancer from 2018 to 2022. We considered distribution of UICC Stages and time from case presentation in our multidisciplinary tumor board or from therapeutic indication from treating physician to therapy initiation (surgery, systemic therapies and radiation) to define delays in diagnosis and treatment.

**Results:**

1020 patients with newly diagnosed lung cancer were referred to our center from 2018 to 2022, with a median of 206 cases yearly (range: 200–208). Cases with Stage IV in 2020–2022 were significantly higher than in 2018–2019 (57% vs. 46%, *p* = 0,001). 228 operative resections took place between 2018 and 2022, 100 from January 2018 to February 2020 and 128 from March 2020 to December 2022. Median time from presentation in our tumor board to resection was also significantly longer after the beginning of the pandemic than before (22 days vs. 15,5 days, *p* = 0,013). No significant delays were observed for administration of systemic treatment and initiation of radiation.

**Conclusions:**

During the pandemic higher disease stages were reported for patients with lung cancer, yet there were no clinically relevant delays in treatment. In the context of the post-covid era new diagnostic strategies are necessary to facilitate early diagnosis of lung cancer. Despite the pandemic, for patients with suspicious symptoms prompt access to healthcare facilities is essential for early diagnosis.

## Background

Lung cancer (LC) remains the leading cause of cancer-related death worldwide in both men and women [1], despite major therapeutic breakthroughs, such as the introduction of Immune-Checkpoint inhibitors (ICI) and Tyrosine-kinase inhibitors (TKIs), which have revolutionized its systemic treatment.

High mortality rates are also due to the lack of a defined screening strategy, especially in patients at high risk for developing LC (e.g. heavy tobacco-smokers, exposure to air pollutant or underlying chronic lung disease), for which most diagnoses occur at an advanced stage, when curative options are limited.

The COVID-19 pandemic has had enormous consequences on the healthcare system worldwide, with about 7 million COVID-19 related deaths having been reported as of today (May 2024) [2].

As most fragile and immunocompromised individuals are more likely to be infected and develop complications from the SARS-CoV-2 virus, cancer patients are regarded as high-risk patients group for Coronavirus-related morbidity and mortality [3]. Amongst patients with solid tumors, LC patients are at particular risk, with mortality rates reported as high as 33% [4], which is the highest reported SARS-CoV-2-related mortality in cancer patients other than in patients with hematological malignancies.

Yet, the consequences of the pandemic on the healthcare system and on cancer patients go far beyond those on individuals infected by the virus.

Since during the most difficult phases of the pandemic many healthcare professionals were directly involved in the treatment of patients affected by SARS-CoV-2, several elective procedures, diagnostic as well as therapeutic ones, had to be postponed. Additionally, fear of infection determined many patients to refer only at later stages to healthcare facilities or their treating physicians even in the presence of alarming symptoms. Consequently, delays in cancer diagnosis and treatment for several tumor entities, including LC, have been reported [5–10].

We hypothesized similar delays at our center, which as a university hospital is both a major regional cancer center as well as one of seven intensive-medicine reference clinics in the federal state of Hessen, Germany.

We retrospectively analyzed data of newly diagnosed Lung Cancer (ND-LC) patients after the outbreak of the COVID-19 pandemic, and from the two preceding years, to determine the degree of delay in cancer diagnosis and treatment in patients referred to our center.

## Methods

### Patients’ selection and data collection

In our analysis we included all patients with ND-LC at any disease stage referred to our center and discussed at our tumor board from January 2018 to December 2022. Patients had to have confirmed histologic diagnosis of either non-small cell lung cancer (NSCLC) or small cell lung cancer (SCLC). We excluded patients with other primary thoracic tumors such as thymoma, thymic carcinoma and carcinoids. We analyzed Union for International Cancer Control (UICC) stages as well as time from case presentation in our tumor board to operation for patients who received partial or total lung resection. Patients who underwent surgery in another center upon presentation in our tumor board were not considered for the latter evaluation.

Time from case presentation or therapeutic indication from referring physician to administration of systemic treatment or initiation of radiation therapy in our center was also analyzed.

To obtain the aforementioned information we screened electronic patient records. All patients’ information was anonymized.

### Data analysis

We analyzed differences between stages at time of referral to our center in the patients’ group 2018–2019 and 2020–2022 as well as for other categorical variables by Fisher’s exact test. The difference between median time from case presentation in the multidisciplinary tumor board to treatment initiation (radiation, surgery, systemic treatment) before and after the pandemic outbreak was analyzed by Mann-Whitney U test. A two-tailed P value < 0,05 was considered as statistically significant.

GraphPad Prism Software, version 8.1.2 (GraphPad Software Inc., San Diego, CA, USA), Jamovi, version 2.3.2 (The jamovi project) and R, version 4.3.2, were used to carry out statistical analyses. Graphs were generated in GraphPad Prism and R.

To analyze the influence of age, sex and histology on time to treatment we performed regression analysis using the “Robustbase” package in R.

## Results

### Patients’ demographics

A total of 1020 patients were referred to our center and diagnosed with lung cancer between 2018 and 2022. 61,6% of patients were male and median age at presentation was 68 (range: 30–91). 80% of patients had NSCLC. Over 50% had metastatic disease and about 65% had a history of smoking (either current or former smoker). Only 5,4% were never smokers and smoking history was not reported for about 29% of patients. Basic demographics of patients’ cohort are summarized in Table 1.

**Table 1:** Patients characteristics of patients referred to our center with ND-LC from 2018 to 2022

	*N*	Percentage (%) or range
Total	1020	
Sex		
Female	628	61,6%
Male	392	38,4%
Age		
	68 (median)	30–91 (range)
Histology		
NSCLC	816	80%
SCLC	204	20%
Stage at presentation		
I	188	18,4%
II	71	7%
III	224	22%
IV	537	52,6%
Smoker status		
Current smoker	401	39,3%
Former smoker	269	26,4%
Never smoker	55	5,4%
Unknown	295	28,9%
Pack years		
	40 (median)*	1-180 (range)*

*this information refers to 608 patients (current and former smokers) for whom data had been collected in patients records

### Disease stages at presentation

In our study we included 1020 patients referred to our center with ND-LC between January 2018 and December 2022. A median of 206 patients were diagnosed each year (range: 200–208). There was no significant increase or decrease in ND-LC cases in the pandemic years (Table 2). 816 (80%) of these patients had NSCLC and 204 (20%) had SCLC, with no significant difference of SCLC diagnoses between the 2018–2019 and the 2020–2022 groups (21 vs. 19,3%, *p* = 0,52).

**Table 2:** Distribution of stages according to diagnosis year and histology

		UICC stages				
Year	Histology	I	II	III	IV	Total
2018	NSCLC	29	17	48	60	154
	SCLC	6	4	11	31	52
2019	NSCLC	44	13	30	80	167
	SCLC	7	1	7	18	33
2020	NSCLC	29	14	42	82	167
	SCLC	7	3	8	23	41
2021	NSCLC	24	7	37	95	163
	SCLC	8	2	8	25	43
2022	NSCLC	32	9	27	97	165
	SCLC	2	1	6	26	35

**Table 3:** Stage I-III vs. IV in the pre-pandemic and pandemic patient group

	Year group			
UICC Stages	2018–2019	2020–2022	Total	Fisher’s exact test
**I-III**	217 (54%)	266 (43%)	483	*p* = 0,001
**IV**	188 (46%)	349 (57%)	537	
	405	615	1020	

**Fig. 1:**
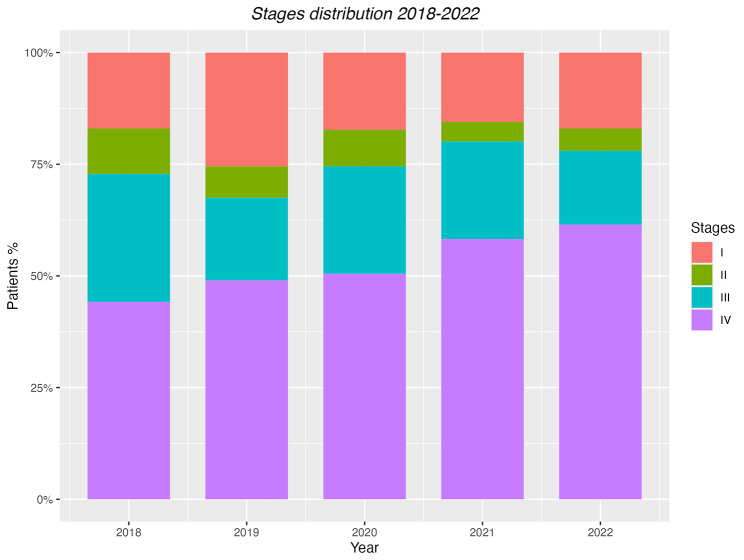
Distribution of disease Stages in ND-LC cases diagnosed at our center

As for disease stages we observed a numerical decrease of all lower tumor stages (I to III). Additionally, the number of patients with stage IV disease was significantly higher in the years 2020–2022 than in 2018–2019 (57% vs. 46%, *p* = 0,001) (see Table 3 Fig. 1).

### Days from case presentation to surgical resection

**Fig. 2:**
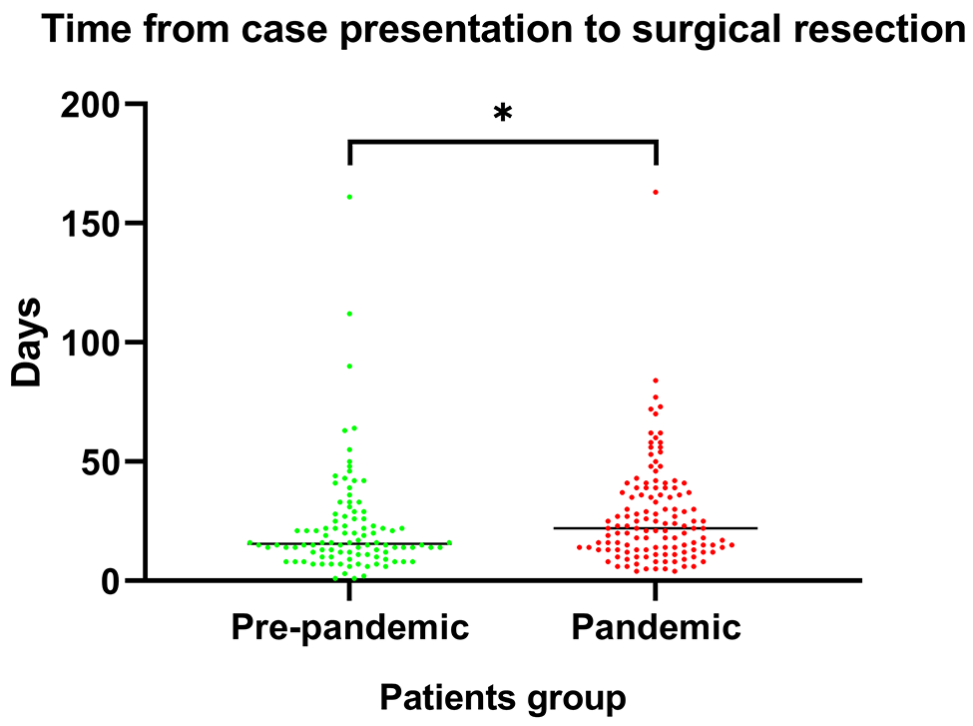
Time from case presentation in our tumor-board to surgery

We evaluated days between case presentation into our multidisciplinary tumor-board and lung resection in patients eligible for surgery. We considered two groups, a pre-pandemic group (January 2018 to February 2020) and a pandemic one (March 2020 to December 2022). Days to surgery in the -pandemic group (median: 22 days, range: 4-163 days) were significantly higher than in the pre-pandemic group (median: 15,5 days, range: 1-161 days) (*p* = 0,013) (Fig. 2).

### Administration of systemic treatment and initiation of radiation therapy

We considered time from therapeutic indication (either upon presentation in the tumor board or by physician’s indication) to administration of all systemic treatments (adjuvant, neoadjuvant, palliative) and radiation therapy when indicated.

173 and 327 patients received systemic treatment in 2018–2019 and 2020–2022 respectively. 164 patients in 2018–2019 and 293 in 2020–2022 had radiation therapy.

There was no difference in median time to systemic treatment in the two groups (23 days for both groups, *p* = 0,83), whereas time to initiation of radiation was significantly shorter during the pandemic (35 days vs. 29 days respectively for 2018–2019 and 2020–2022, *p* = 0,012).

### Influence of histology, age and sex on time to treatment

We analyzed the influence of histology, age and sex on time to different treatments using regression model. Given the violation of normality of data and residuals we performed a robust regression (see materials and methods). Small cell histology was associated with significantly shorter time to administration of systemic treatment. This was confirmed using univariate analysis, with a median time to systemic treatment of 4,5 days and 29 days for SCLC and NSCLC respectively (*p* < 0,001, Mann-Whitney U test).

Histology affected neither time to surgery nor time to radiation therapy in our patient population. Age and sex did not affect therapy.

## Discussion

Our single-center analysis confirms data from other studies on LC and other malignancies showing a tendency towards diagnosis of higher disease stages in ND-LC after the outbreak of the COVID-19-pandemic [5, 6, 8]. Additionally, consistent with other reports, we observed a significant increase in the time to case presentation in our interdisciplinary tumor board and lung resection in operable cases (22 vs. 15,5 days before the pandemic) [11].

Different reasons might explain the observed diagnostic delays. First, some warning signs and symptoms in LC, such as cough and fatigue, may mimic those of a respiratory infection. Particularly, in the case of co-occurring SARS-CoV-2 infection those symptoms may initially just be attributed to the infection. As persistence of these symptoms has been reported also after recovery from infection, these may not be regarded as an alarming sign by patients or their treating physicians. Additionally, a general fear of infection on the patients’ side as well as more limited access to primary care facilities may have caused late admission in hospitals and clinics, therefore delaying diagnoses and treatment [12].

As for treatment delays, median time from case presentation to surgical resection was about one week longer in the pandemic years compared to the 2 years before. We consider one of the main reasons for this delay to be the lower availability of Intermediate care unit (IMCU) and intensive care unit (ICU) beds during the pandemic. Indeed, planning for every elective lung surgery includes also foreseeing to manage possible post-surgical complications, which may require specific care in IMCU or ICU. Yet, while this difference was statistically significant, we do not consider it to be clinically meaningful, as this time, even if prolonged for our standards, was still in line with that of previous reports [13–15].

Conversely, timely start of systemic treatment and radiation therapy was possible even during the pandemic, as no significant delays were observed. This is in part in contrast with other publications showing important delays in administration of systemic therapies in cancer patients [16].

Interestingly, unlike other studies, we did not observe a drop in the total number of ND-LC cases in the first pandemic year, nor later [17]. This shows that despite the difficulties posed by the pandemic, similar access to our center’s facilities was available to patients.

While our data shows an increase of diagnoses at later stages during and after the pandemic, even before the pandemic most patients were diagnosed at advanced stages. The presence of the pandemic adds therefore a secondary challenge to a problem that was already present. In this sense, awareness of differential diagnosis of LC with SARS-CoV-2 infection and post-covid disease is of the utmost importance. The presence of alarming signs or symptoms, such as persisting cough, hemoptysis, and weight loss, should always be seen as suspicious, regardless of a previous or concurrent SARS-CoV-2 infection, especially in patients at higher risk of developing LC. In the future the introduction of new guidelines or screening strategies [18] might be helpful in guiding primary care physicians’ decision-making and defining when further diagnostics is necessary.

Importantly, patients with suspicious symptoms should be immediately referred to specialized healthcare facilities, regardless of the pandemic.

## Data Availability

The datasets used and/or analyzed during the current study are available from the corresponding author on reasonable request.

## Abbreviations

COVID-19: Coronavirus Disease 19
ICI: Immune-checkpoint inhibitor
ICU: Intensive care unit
IMCU: Intermediate care unit
LC: Lung cancer
ND-LC: Newly diagnosed lung cancer
NSCLC: Non-small cell lung cancer
SARS-CoV-2: Severe acute respiratory syndrome coronavirus 2
SCLC: Small-cell lung cancer
TKI: Tyrosine-kinase inhibitor
UICC: Union for International Cancer Control

## References

[1] Lung cancer. Accessed: Oct. 30. 2023. [Online]. Available: https://www.who.int/news-room/fact-sheets/detail/lung-cancer.

[2] WHO Coronavirus (COVID-19). Dashboard. Accessed: Oct. 30, 2023. [Online]. Available: https://covid19.who.int.

[3] Bora VR, Patel BM. The deadly duo of COVID-19 and Cancer! Front Mol Biosci. 2021;8. 10.3389/fmolb.2021.643004. Frontiers Media S.A. 10.3389/fmolb.2021.643004 PMC8072279 33912588

[4] Garassino MC, et al. COVID-19 in patients with thoracic malignancies (TERAVOLT): first results of an international, registry-based, cohort study. Lancet Oncol. Jul. 2020;21(7):914–22. 10.1016/S1470-2045(20)30314-4. 10.1016/S1470-2045(20)30314-4 PMC7292610 32539942

[5] Cantini L, et al. Evaluation of COVID-19 impact on DELAYing diagnostic-therapeutic pathways of lung cancer patients in Italy (COVID-DELAY study): fewer cases and higher stages from a real-world scenario. ESMO Open. Apr. 2022;7(2). 10.1016/j.esmoop.2022.100406. 10.1016/j.esmoop.2022.100406 PMC8810307 35219245

[6] Fanning JE, Kalsi S, Krag DN. Impact of the COVID-19 pandemic on melanoma diagnosis and presentation: a `review, *International Journal of Dermatology*, vol. 62, no. 7. John Wiley and Sons Inc, pp. 850–856, Jul. 01, 2023. 10.1111/ijd.16684. 10.1111/ijd.16684 37073701

[7] Greene G et al. Aug., Impact of the SARS-CoV-2 pandemic on female breast, colorectal and non-small cell lung cancer incidence, stage and healthcare pathway to diagnosis during 2020 in Wales, UK, using a national cancer clinical record system, *Br J Cancer*, vol. 127, no. 3, pp. 558–568, 2022, 10.1038/s41416-022-01830-6. 10.1038/s41416-022-01830-6 PMC9060409 35501391

[8] Park JY, et al. Collateral effects of the coronavirus disease 2019 pandemic on lung cancer diagnosis in Korea. BMC Cancer. Dec. 2020;20(1). 10.1186/s12885-020-07544-3. 10.1186/s12885-020-07544-3 PMC7594984 33121456

[9] Terashima T, Tsutsumi A, Iwami E, Kuroda A, Nakajima T, Eguchi K. Delayed visit and treatment of lung cancer during the coronavirus disease 2019 pandemic in Japan: a retrospective study. J Int Med Res. May 2022;50(5). 10.1177/03000605221097375. 10.1177/03000605221097375 PMC9128063 35579175

[10] Reyes R et al. MA03.08 Impact of COVID-19 Pandemic in the Diagnosis and Prognosis of Lung Cancer, *Journal of Thoracic Oncology*, 2021, Accessed: Oct. 30, 2023. [Online]. Available: 10.1016/j.jtho.2021.01.219.

[11] Tomos I, et al. The impact of COVID-19 pandemic on Surgical Treatment of Resectable Non-small Cell Lung Cancer in Greece. Life. Jan. 2023;13(1). 10.3390/life13010218. 10.3390/life13010218 PMC9864579 36676167

[12] Bakhribah H et al. Implications of COVID-19 pandemic on lung cancer management: A multidisciplinary perspective, *Critical Reviews in Oncology/Hematology*, vol. 156. Elsevier Ireland Ltd, Dec. 01, 2020. 10.1016/j.critrevonc.2020.103120. 10.1016/j.critrevonc.2020.103120 PMC7546967 33099232

[13] Labbé C (2017). Wait times for diagnosis and treatment of lung cancer: a single-centre experience. Curr Oncol.

[14] Vidaver RM, Shershneva MB, Hetzel SJ, Holden TR, Campbell TC. Typical time to treatment of patients with lung cancer in a multisite, US-based study. J Oncol Pract. Jun. 2016;12(6):e643–51. 10.1200/JOP.2015.009605. 10.1200/JOP.2015.009605 27143146

[15] Maiga AW (2017). Timeliness of Care and Lung Cancer Tumor-Stage Progression: how long can we wait?. Ann Thorac Surg.

[16] de Joode K et al. Sep., Impact of the coronavirus disease 2019 pandemic on cancer treatment: the patients’ perspective, *Eur J Cancer*, vol. 136, pp. 132–139, 2020, 10.1016/j.ejca.2020.06.019. 10.1016/j.ejca.2020.06.019 PMC7334940 32683273

[17] Kasymjanova G et al. Nov., The Impact of COVID-19 on the Diagnosis and Treatment of Lung Cancer over a 2-Year Period at a Canadian Academic Center, *Curr Oncol*, vol. 29, no. 11, pp. 8677–8685, 2022, 10.3390/curroncol29110684. 10.3390/curroncol29110684 PMC9689557 36421337

[18] Mazzone PJ, et al. Management of lung nodules and Lung Cancer Screening during the COVID-19 pandemic: CHEST Expert Panel Report. Chest. Jul. 2020;158(1):406–15. 10.1016/j.chest.2020.04.020. 10.1016/j.chest.2020.04.020 PMC7177089 32335067

